# COVID-19 and spontaneous pneumomediastinum: a rare complication

**DOI:** 10.1590/0037-8682-0871-2020

**Published:** 2021-02-26

**Authors:** Júlio Holanda Cavalcanti de Albuquerque, Angélica Maria Holanda Pascoal da Silva, Tássia Ívila Freitas de Almeida, Luís Arthur Brasil Gadelha Farias

**Affiliations:** 1 Hospital Geral de Fortaleza, Serviço de Clínica Médica, Fortaleza, CE, Brasil.; 2 Hospital São José de Doenças Infecciosas, Fortaleza, CE, Brasil.

**FIGURE 1:**
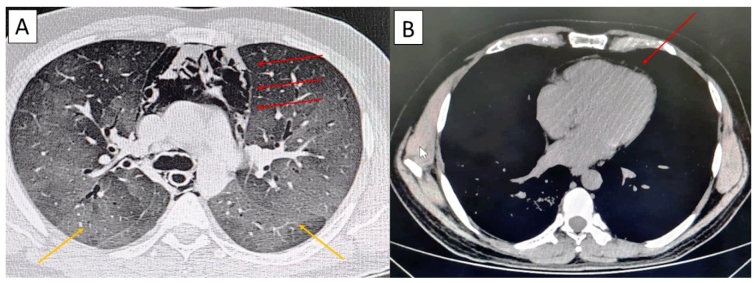
Axial unenhanced chest computed tomography. **(A)** Large pneumomediastinum (red arrows) and multiple ground-glass opacities (yellow arrows) in both lung fields. **(B)** Resorption of the pneumomediastinum with a small layer of air (red arrow).

A 42-year-old man presented with a 9-day history of fever (39°C), cough, odynophagia, myalgia, and dyspnea. His symptoms had worsened 2 days prior to presentation. Physical examination revealed mild respiratory distress-respiratory rate, 35 rpm; heart rate, 140 bpm; and blood oxygen saturation level without supplementary oxygen, 91%. Chest examination revealed reduced vesicular breath sounds in both lung bases. On admission, he had normal blood cell, platelet, and leukocyte counts; lymphopenia (lymphocyte count: 676/mm^3^); high C-reactive protein level (391.35 mg/L); normal hepatic and renal functions; and normal D-dimer, troponin, aspartate transaminase, alanine transaminase, lactate dehydrogenase, and ferritin levels. Chest computed tomography (CT) revealed spontaneous pneumomediastinum and bilateral ground-glass opacities (Figure 1A). He was diagnosed with coronavirus disease (COVID-19) using reverse-transcription polymerase chain reaction analysis of a nasopharyngeal swab specimen and received a 5-day regimen of methylprednisolone (40 mg/day), hydroxychloroquine (400 mg/day), azithromycin (500 mg/day), and ceftriaxone (2 g/day). During hospitalization, he was weaned of oxygen support and discharged after 10 days. Chest CT on discharge revealed resorption of the pneumomediastinum (Figure 1B).

Spontaneous pneumomediastinum is usually self-limiting, as in this case. Thus, only a few COVID-19-related pneumomediastinum cases with fatal outcomes have been reported^1^. An emerging pressure gradient between the alveoli and surrounding structures may cause alveolar rupture, with air leaking into the interstitium, following the bronchovascular bundle toward the hilum and spreading through the mediastinum^2^ ^,^ ^3^. Although this is a rare manifestation of COVID-19, physicians in pandemic settings should be aware of this complication.
